# Synthetic analysis of associations between IL-10 polymorphisms and skin cancer risk

**DOI:** 10.18632/oncotarget.23385

**Published:** 2017-12-17

**Authors:** Hongbo Zhao, Jiaoli Yang, Zhenzhen Yu, Hui Shen, Xinlin Huang, Mi Zhang, Teng Long, A Cailing, Wenhui Wang

**Affiliations:** ^1^ Department of Dermatology, Renmin Hospital, Hubei University of Medicine, Shiyan, Hubei 442000, China; ^2^ Department of Traditional Chinese Medicine, Renmin Hospital, Hubei University of Medicine, Shiyan, Hubei 442000, China

**Keywords:** IL-10, skin cancer, polymorphism, meta-analysis

## Abstract

The current study was designed to quantitatively summarize the evidence for the strength of the associations between common IL-10 functional polymorphisms and skin cancer risk. Relevant publications concerning the associations between common IL-10 functional polymorphisms(−1082G>A, −819C>T and −592C>A) and skin cancer were retrieved by a comprehensive electronic literature search in PubMed, Web of Science, EBSCO, Embase, China National Knowledge Infrastructure, Wanfang, Chinese Biomedical Database (CBM). The odds ratio (OR) and 95% confidence interval (CI) were utilized to assess the strength of the relationship. A total of 26 studies including 4090 cases and 4133 controls (−1082G>A, 10 studies with 1809 cases and 1830 controls; −819C>T, 7 studies with 862 cases and 957 controls; −592C>A, 9 studies with 1419 cases and 1346 controls) were enrolled in the meta-analysis. Overall, the results revealed a borderline decreased risk of skin cancer in heterozygote model (OR = 0.82, 95CI = 0.67–1.00, *p =* 0.05). The subgroup analysis also presented similar association for non-melanoma skin cancer in heterozygote model (OR = 0.67, 95CI = 0.50–0.91, *p =* 0.01). Moreover, the further analysis based on the histological type of non-melanoma skin cancer indicated a significantly decreased risk of BCC in allele model (OR = 0.67, 95% CI = 0.50–0.91, *p =* 0.02) and dominant model (OR = 0.68, 95% CI = 0.48–0.98, *p =* 0.04). However, neither overall analysis nor subgroup analysis based on cancer subtype revealed a significant association of −1082G>A or −592C>A polymorphisms with skin cancer. The present study suggested a potential association between IL-10 −819C>T polymorphism and decreased risk of skin cancer, but a lack of association for −1082G>A and −592C>A polymorphisms. Further invalidation is urgently needed.

## INTRODUCTION

Skin cancer classified as either non-melanoma and melanoma dermatic carcinoma represents one of the most common malignancies in white-skinned populations, with increasing incidence in recent years [1, 2]. Despite of the extensively observed threatens on lifetime and wholesome appearance for affected individuals especially for melanoma patients, the pathogenesis of skin cancer is multifactorial, complicated and not fully understood as yet, and relatively little is known about the genetic factors mediating susceptibility to skin cancer in addition to the well-known risk factors including sunlight exposure, age, family history of cancer, dysplastic or atypical nevi and skin color [3–5]. The ongoing investigations tend to focus on the molecular basis of genetic heterogeneity in relation to genes involved in immune reaction, inflammation, DNA repair and etc. [6]. Recently, emerging evidence has revealed the crucial role of the imbalance of anti-tumor immune response and immunosuppressive effect mediated by cytokines in the development of skin cancer [7–9].

Interleukin-10 (IL-10), as an important immuno-modulator with pleiotropic functions, is produced by multiple cell types including keratinocytes, immunocyte and tumor cells [10, 11]. Although IL-10 was documented to exert anti-tumor and antimetastatic activity by inhibiting angiogenesis by numerous lines of evidence which was further supported by the observed delay in progression or complete regression of tumor in murine models with the administration of recombinant human IL-10 [12, 13], the production of IL-10 by keratinocytes or tumor infiltrating cells has also been found to exhibit cancer-promoting ability by inducing cell proliferation and down-modulating the anti-tumor immune response in skin cancer [14]. In support of the cancer-promoting role, elevated expression of IL-10 was observed in many cancers, including non-melanoma and melanoma skin cancer [15]. Mechanistically, IL-10 has been found to be implicated in inhibition of the T-cell immune response and can act as an autocrine growth factor for melanoma cells, down-regulating the expression of HLA molecules to contribute to the immune escape [16].

*In vitro* studies showed striking differences in the ability to produce IL-10 between individuals following mitogen stimulation of whole-blood cultures, which mainly appears in different efficiency of IL-10 mRNA synthesis [17]. The different individual capacity to synthesize IL-10 seems to be genetically controlled [18]. Genetic variations in IL-10 gene may lead to different immune responses and susceptibility to skin cancer [19]. Although a few studies have examined the relationship between common IL-10 polymorphisms and susceptibility to skin cancer, inconsistent results were obtained potentially due to a limited sample size or histological variations. In the current study, we sought to give a more authentic cognition concerning the associations between the common IL-10 polymorphisms (−1082G>A, −819C>T and −592C>A) and susceptibility to skin cancer by a meta-analysis.

## RESULTS

### Characteristics of eligible studies

**Figure 1:**
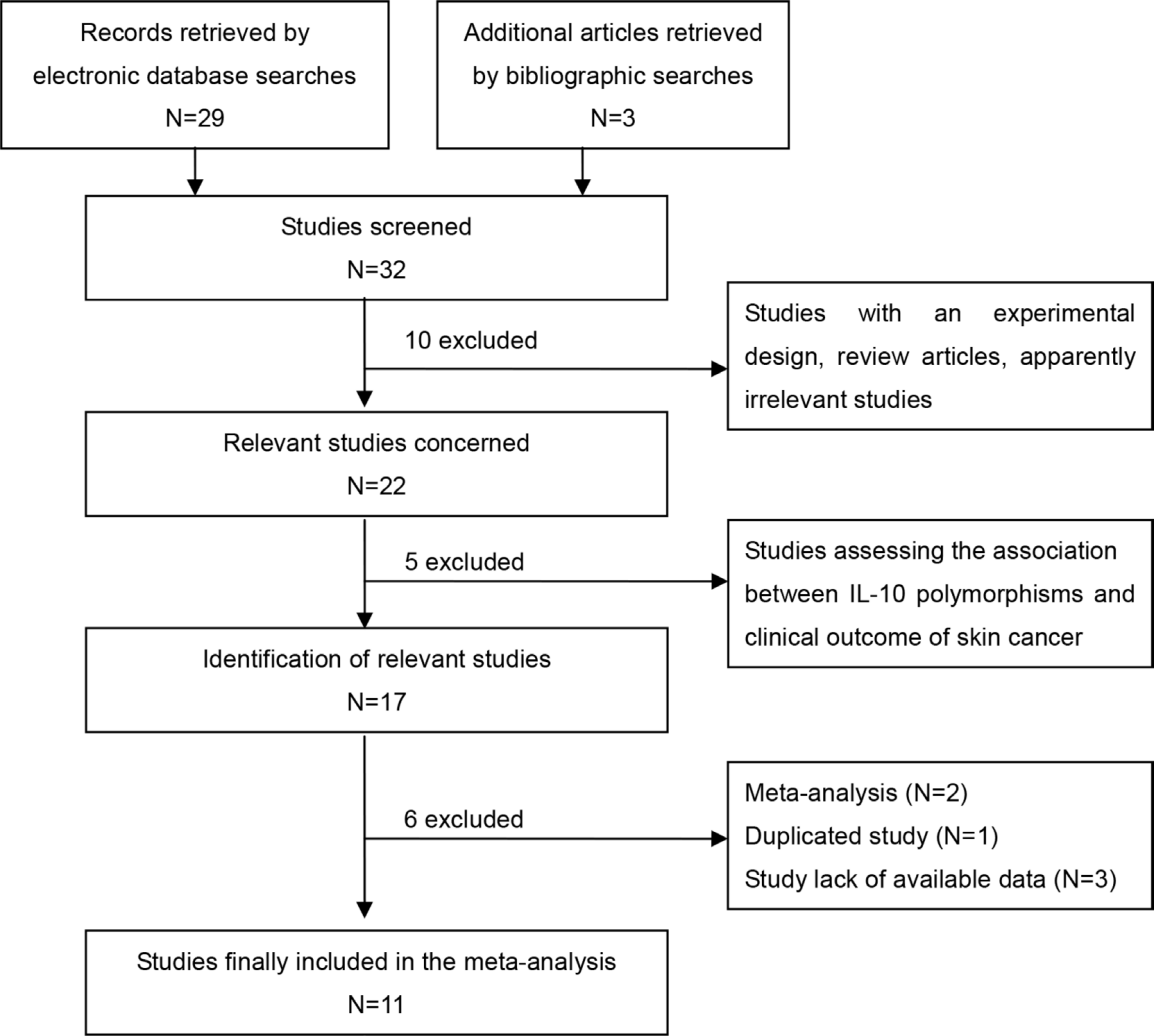
Flow chart of selection procedure.

Detailed flow of study selection is displayed in Figure 1. A total of 32 articles were retrieved by the initial search. Finally, 26 individual studies from 11 publications assessing the associations between the three common IL-10 polymorphisms(−1082G>A, −819C>T and −592C>A) and skin cancer risk were enrolled in the meta-analysis subsequent to a careful review of the titles, abstracts and full text based on the defined criteria [6, 20–29]. All the studies were population-based and conducted in Caucasian population, resulting in 4090 cases and 4133 controls (−1082G>A, 1809 cases and 1830 controls; −819C>T, 862 cases and 957 controls; −592C>A, 1419 cases and 1346 controls). Of which, there were 10, 7, and 9 case-control studies for the −1082G>A, −819C>T and −592C>A polymorphisms, respectively. The distribution of genotypes in the controls of all included studies was in agreement with Hard-Weinberg equilibrium (HWE) except for the studies by Festa *et al*. [23] and the study by Howell *et al*. [26] on −1082G>A. Detailed characteristics of the included studies are summarized in Table 1.

**Table 1:** Characteristics of included studies

First author	Year	Country	Ethnicity	Genotype-case	Genotype-control	Source of control	Genotype method	Cancer type	IL-10 polymorphism	HWE test
				VR Ho/Ht/WT Ho	VR Ho/Ht/WT Ho					
Alonso [20]	2005	Spain	Caucasian	39/43/16	42/42/16	population	PCR-RFLP	melanoma	−1082G>A	0.32
	2005	Spain	Caucasian	6/40/52	11/36/53	population	PCR-RFLP	melanoma	−819C>T	0.21
	2005	Spain	Caucasian	6/40/52	11/36/53	population	PCR-RFLP	melanoma	−592C>A	0.21
Martínez-Escribano [21]	2002	Spain	Caucasian	15/22/5	19/21/8	population	PCR-RFLP	melanoma	−1082G>A	0.60
	2002	Spain	Caucasian	1/18/23	4/17/27	population	PCR-RFLP	melanoma	−819C>T	0.58
	2002	Spain	Caucasian	1/18/23	4/17/27	population	PCR-RFLP	melanoma	−592C>A	0.58
Nikolova [22]	2007	Mixed	Caucasian	52/38/30	37/58/25	population	PCR-SSP	SCC	−1082G>A	0.80
	2007	Mixed	Caucasian	22/34/64	8/45/67	population	PCR-SSP	SCC	−819C>T	0.90
	2007	Mixed	Caucasian	22/34/64	8/45/67	population	PCR-SSP	SCC	−592C>A	0.90
Festa [23]	2005	Sweden	Caucasian	69/97/75	82/110/68	population	PCR-RFLP	BCC	−1082G>A	0.01
	2005	Sweden	Caucasian	18/78/145	13/103/44	population	PCR-RFLP	BCC	−592C>A	<0.01
Wilkening [24]	2006	Germany	Caucasian	104/255/167	89/264/175	population	Taqman	BCC	−1082G>A	0.53
Sobjanek [6]	2015	Poland	Caucasian	30/140/9	36/90/47	population	SSP-PCR	BCC	−1082G>A	0.56
	2015	Poland	Caucasian	10/51/111	18/96/143	population	SSP-PCR	BCC	−819C>T	0.73
Schoof [25]	2009	Germany	Caucasian	52/76/37	39/89/34	population	Taqman	melanoma	−1082G>A	0.2
	2009	Germany	Caucasian	16/59/89	8/56/98	population	Taqman	melanoma	−592C>A	1.00
Howell [26]	2001	UK	Caucasian	41/72/40	27/92/39	population	ARMS-PCR	melanoma	−1082G>A	0.03
	2001	UK	Caucasian	7/45/98	8/59/91	population	ARMS-PCR	melanoma	−819C>T	0.69
	2001	UK	Caucasian	12/59/94	8/59/91	population	ARMS-PCR	melanoma	−592C>A	0.69
Gu [27]	2008	USA	Caucasian	46/112/57	44/103/64	population	OpenArray™	melanoma	−1082G>A	0.83
	2008	USA	Caucasian	11/84/115	7/83/114	population	OpenArray™	melanoma	−819C>T	0.08
	2008	USA	Caucasian	9/83/121	9/80/124	population	OpenArray™	melanoma	−592C>A	0.38
Alamartine [28]	2003	France	Caucasian	16/37/17	24/33/13	population	PCR-RFLP	SCC and BCC	−1082G>A	0.78
	2003	France	Caucasian	1/19/50	8/27/35	population	PCR-RFLP	SCC and BCC	−819C>T	0.43
	2003	France	Caucasian	1/19/50	8/27/35	population	PCR-RFLP	SCC and BCC	−592C>A	0.43
Vogel [29]	2007	Denmark	Caucasian	17/102/185	15/106/194	population	PCR-RFLP	BCC	−592C>A	0.92

BCC, basal cell carcinoma; SCC, squamous cell carcinoma; VR, variant; WT, wild-tlype; Ht, heterozygote; VR Ho, variant homozygote; WT Ho, wide-type homozygote; HWE, Hardy-Weinberg equilibrium.

### Quantitative synthesis

### IL-10 −1082G>A

**Figure 2:**
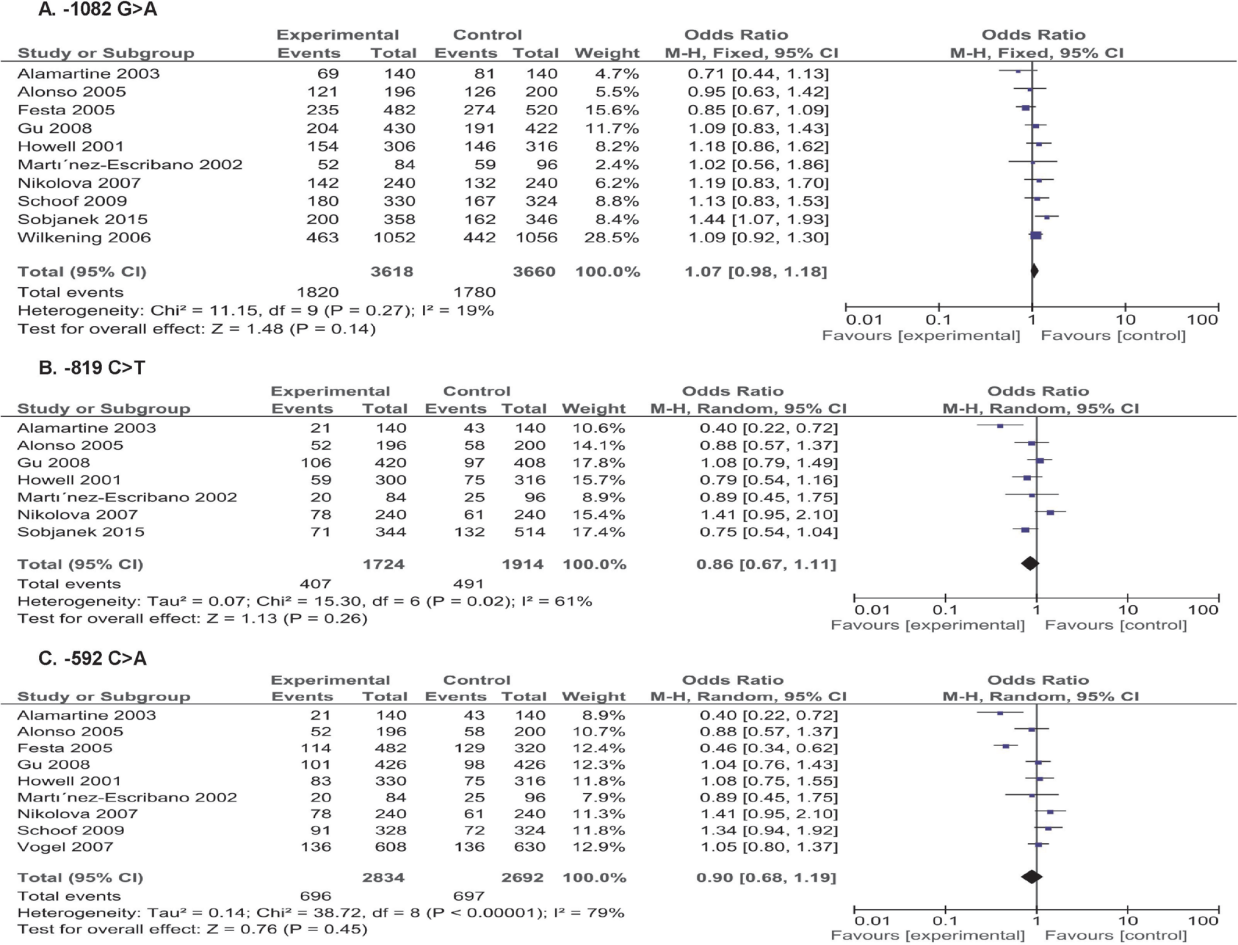
Forest plots of skin cancer risk associated with IL-10 gene −1082G>A, −819C>T and −592C>A polymorphisms (**A–C**) in the allele contrast (A vs. G; T vs. C; A vs. C, respectively).

The aggregated ORs and heterogeneity test results for the association between the −1082G>A polymorphism and the risk of skin cancer are presented in Table 2 and Figure 2. As a result, 10 studies with a total of 1809 cases and 1830 controls were included in the study. Overall, no significant association was found under any genetic model (all *p >* 0.05). Similarly, there was no significant association revealed in the melanoma and non- melanoma skin cancer subgroup analyses (all *p >* 0.05). Moreover, a further subgroup analysis according to the histological type of non-melanoma skin cancer showed no significant relationship whatever in basal cell carcinoma (BCC) and squamous cell carcinoma (SCC, all *p >* 0.05). The results are detailed in Table 2.

**Table 2:** Meta-analysis results of association between IL-10 polymorphisms and skin cancer risk

IL-10 variants	Cancer type	Studies	Allele contrast		Ht vs. WT Ho		VR Ho vs. WT Ho		Dominant model		Recessive model	
			OR [95%CI]	*P_h_*/I^2^	OR [95%CI]	*P_h_*/I^2^	OR [95%CI]	*P_h_*/I^2^	OR [95%CI]	*P_h_*/I^2^	OR [95%CI]	*P_h_*/I^2^
−1082G>A	overall	10	1.07 [0.98, 1.18]	0.27/19%	1.09 [0.76, 1.57]	<0.001/76%	1.17 [0.89, 1.54]	0.07/44%	1.12 [0.82, 1.52]	0.0004/70%	1.10 [0.95, 1.28]	0.11/37%
	melanoma	5	1.09 [0.94, 1.27]	0.94/0%	0.98 [0.75, 1.28]	0.55/0%	1.20 [0.88, 1.64]	0.94/0%	1.05 [0.81, 1.35]	0.87/0%	1.21 [0.95, 1.54]	0.35/11%
	NMC	5	1.05 [0.85, 1.29]	0.04/61%	1.22 [0.61, 2.41]	<0.001/88%	1.17 [0.70, 1.97]	0.005/73%	1.22 [0.68, 2.22]	<0.001/86%	1.00 [0.73, 1.37]	0.06/56%
	BCC	4	1.07 [0.86, 1.33]	0.06/59%	1.54 [0.67, 3.52]	<0.001/90%	1.31 [0.70, 2.46]	0.006/76%	1.47 [0.70, 3.10]	<0.001/89%	1.00 [0.81, 1.24]	0.39/1%
	SCC	2	1.03 [0.76, 1.40]	0.16/49%	0.66 [0.38, 1.14]	0.33/0%	0.62 [0.15, 2.58]	0.05/75%	0.76 [0.46, 1.26]	0.80/0%	0.73 [0.12, 4.41]	0.002/89%
−819C>T	overall	7	0.86 [0.67, 1.11]	0.02/61%	**0.82 [0.67, 1.00]**	0.43/0%	0.83 [0.42, 1.64]	0.02/59%	0.84 [0.69, 1.01]	0.19/31%	0.89 [0.45, 1.74]	0.02/60%
	melanoma	4	0.93 [0.76, 1.14]	0.64/0%	0.95 [0.73, 1.23]	0.53/0%	0.84 [0.48, 1.47]	0.40/0%	0.93 [0.72, 1.19]	0.64/0%	0.84 [0.49, 1.46]	0.34/11%
	NMC	3	0.77 [0.41, 1.45]	0.001/85%	**0.67 [0.50, 0.91]**	0.60/0%	0.74 [0.16, 3.41]	0.004/82%	0.70 [0.43, 1.16]	0.06/64%	0.87 [0.20, 3.75]	0.005/81%
	BCC	2	**0.72 [0.54, 0.97]**	0.60/0%	0.71 [0.48, 1.03]	0.74/0%	0.58 [0.28, 1.22]	0.29/12%	**0.68 [0.48, 0.98]**	0.94/0%	0.66 [0.32, 1.36]	0.26/20%
	SCC	2	0.74 [0.19, 2.83]	0.001/90%	0.68 [0.43, 1.09]	0.36/0%	0.57 [0.01, 26.7]	0.01/85%	0.68 [0.24, 1.94]	0.03/78%	0.68 [0.02, 26.5]	0.01/83%
−592C>A	overall	9	0.90 [0.68, 1.19]	<0.001/79%	0.80 [0.55, 1.16]	<0.001/80%	0.95 [0.54, 1.66]	0.006/62%	0.83 [0.57, 1.20]	<0.001/82%	1.10 [0.68, 1.77]	0.04/51%
	melanoma	5	1.08 [0.90, 1.28]	0.62/0%	1.06 [0.84, 1.32]	0.99/0%	1.15 [0.73, 1.80]	0.24/27%	1.09 [0.88, 1.35]	0.94/0%	1.11 [0.71, 1.72]	0.21/32%
	NMC	4	0.73 [0.41, 1.30]	<0.001/90%	0.55 [0.26, 1.17]	<0.001/89%	0.77 [0.26, 2.29]	<0.001/81%	0.59 [0.27, 1.27]	<0.001/91%	1.09 [0.46, 2.60]	0.02/71%
	BCC	3	0.67 [0.37, 1.24]	<0.001/87%	0.56 [0.20, 1.63]	<0.001/93%	0.60 [0.24, 1.51]	0.09/59%	0.55 [0.20, 1.52]	<0.001/93%	0.92 [0.56, 1.49]	0.34/7%
	SCC	2	0.74 [0.19, 2.83]	0.001/90%	0.68 [0.43, 1.09]	0.36/0%	0.57 [0.01, 26.7]	0.01/85%	0.68 [0.24, 1.94]	0.03/78%	0.68 [0.02, 26.5]	0.01/83%

NMC, non-melanoma cancer; BCC, basal cell carcinoma; SCC, squamous cell carcinoma; VR, variant; WT, wild-type; Ht, heterozygote; VR Ho, variant homozygote; WT Ho, wide-type homozygote; *P_h_* , *P* value for heterogeneity test.

### IL-10 −819C>T

The −819 C>T polymorphism was investigated in 7 studies with a total of 862 cases and 957 controls. As shown in Table 2 and Figure 2, the overall analysis revealed a borderline decreased risk of skin cancer in heterozygote model (OR = 0.82, 95CI = 0.67–1.00, *p* = 0.05). The subgroup analysis also presented a reduced skin cancer risk associated with −819 C>T variant of IL-10 in heterozygote model (OR = 0.67, 95CI = 0.50–0.91, *p* = 0.01) in non-melanoma skin cancer but not in melanoma skin cancer in any genetic model (all *p >* 0.05). Moreover, the further analysis based on the histological type of non-melanoma skin cancer indicated a significantly decreased risk of BCC in allele model (OR = 0.67, 95% CI = 0.50–0.91, *p* = 0.02) and dominant model (OR = 0.68, 95% CI = 0.48–0.98, *p* = 0.04) but not in melanoma skin cancer (Table 2).

### IL-10 −592C>A

Nine studies involving 1419 cases and 1346 controls were pooled into the meta-analysis to investigate the association concerning −592C>A polymorphism. Overall, as shown in Table 2 and Figure 2, there were no significant association observed in any genetic model (all *p >* 0.05). Furthermore, no significant association was observed in the melanoma and non- melanoma skin cancer subgroup analyses in any genetic model (all *p >* 0.05). Also, no significant association concerning the −592C>A and the risk of melanoma skin cancer was suggested whatever in BCC and SCC (all *p >* 0.05).

### Publication bias and sensitivity analysis

**Figure 3:**
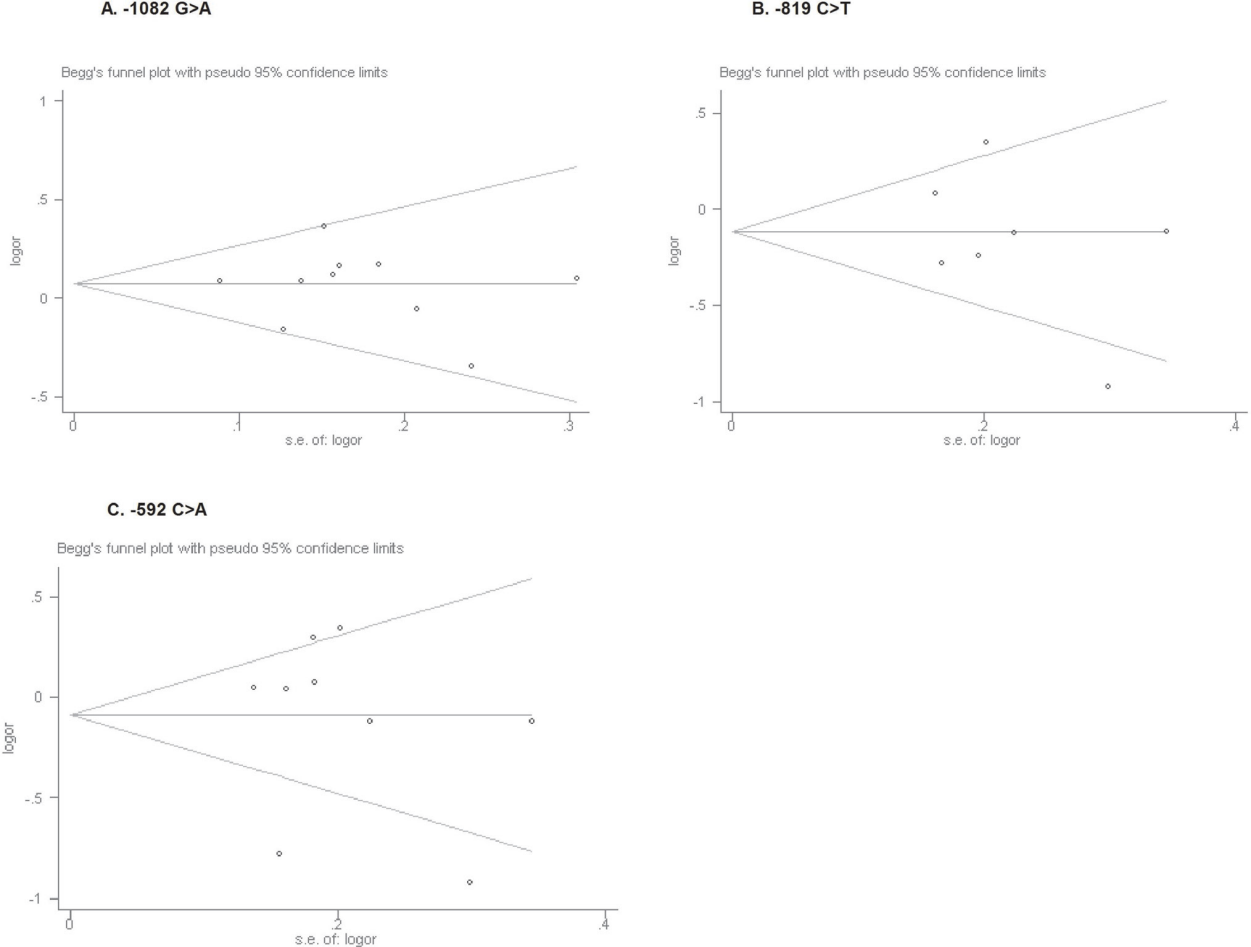
Begg's funnel plots for publication bias test on the associations of IL-10 gene −1082 G>A, −819C>T and −592C>A polymorphisms (**A–C**) with skin cancer risk in the allele contrast (A vs. G; T vs. C; A vs. C, respectively).

Begg's funnel plot and Egger's linear regression test were performed to assess the potential publication bias. As observed in Figure 3, no obvious asymmetry was presented on the Begg's funnel plot, indicating no significant publication bias, which was further supported by the Egger's test (allele model: *p* = 0.736 for −1082G>A; *p* = 0.392 for −819C>T; *p* = 0.729 for −592C>A). Sensitivity analysis by sequential omission of each individual study revealed no substantial alterations of the synthetic results for −1082G>A and −592C>A polymorphisms, suggesting the robustness and reliability of results. However, we found a slight fluctuation of pooled results regarding −819C>T polymorphism under dominant and heterozygote models (shown in Supplementary Table 1), implying the potential source of heterogeneity and caution to interpret the results.

## DISCUSSION

The present study based on 26 studies including 4090 cases and 4133 controls was the first article employing a meta-analysis to specify the effect of common IL-10 polymorphisms on susceptibility to skin cancer. We found that neither −1082G>A nor −592C>A was a conspicuous low-penetrant risk factor for developing skin cancer, However, the results indicated that IL-10 −819C>T variant may contribute, although modestly, to the reduction of skin cancer risk, especially for BCC subtype.

Given the importance of IL-10 protein which is a multifunctional cytokine with both immunosuppressive and anti-angiogenic functions in cancers [10], a genetic predisposition concentrating on IL-10 common variants to skin cancer has been suggested by a growing of studies [30]. Variants in IL-10 have been detected at several loci, and the three most common polymorphisms (−1082G>A, −819C>T and −592C>A) located in the promoter of IL-10 have been reported to be capable of influencing the transcription and translation of IL-10 gene, resulting in altered susceptibility and prognosis of skin cancer [20, 31, 32]. In these regards, *in-vitro* studies have revealed that individuals with −1082 A, −819 T, or −592 A carrier accompany generally with decreased IL-10 production compared to corresponding −1082 G, −819 C, or −592 C carrier, respectively [33]. Up to now, a number of studies have been performed to evaluate the effect of common IL-10 polymorphisms on the risk of skin cancer. However, most of these studies were based on a small sample size, which may lead to a distorted impression of the genetic etiology of skin cancer. Moreover, results from previous individual studies have constantly remained inconsistent even conflicting. Thus, a systematic meta-analytic approach which may assist in estimating population-wide effects of genetic risk factors in human disease was performed to reassess the concerned associations.

In the present study, a potential role of IL-10 −819C>T polymorphism, although modest, in reducing the risk of skin cancer was revealed by pooling 7 individual studies with 862 cases and 957 controls, suggesting that TT genotype may be more protective from the susceptibility to skin cancer. Moreover, we also observed a marginal significance of TT+CT genotype associated with a decreased risk of skin caner. The mechanism accounting for these findings remains largely undetermined, but may likely be implicated in the regulation of IL-10 expression and attenuation of tumor escape by IL-10-mediated immunosuppression [33]. Whereas, results from most of previous studies were not in accordance with our findings, suggesting the potential significance of sample size in determining the strength of the association. Moreover, the stratification analysis by cancer subtype also supported a possible protective role of T-carrier in the risk reduction of non-melanoma cancer, especially basal cell carcinoma, but not for melanoma, suggesting that the histological specificity of skin cancer may likely be significant for the assessment regarding the association. Nevertheless, these findings are greatly needed to be further confirmed due to limited studies enrolled in the stratification analysis which may lower the statistical power.

For the IL-10 −1082G>A polymorphism, 10 studies with 1809 cases and 1830 controls were pooled into the analysis. Neither overall analysis nor subgroup analysis based on cancer subtype revealed a significant association of −1082G>A polymorphism with skin cancer. Similarly, no significant association for skin cancer risk was observed regarding IL-10 −592C>A polymorphism with a total of 1419 cases and 1346 controls. Although it is biologically plausible that IL-10 −1082G>A and −592C>A polymorphisms which are both able to affect IL-10 expression [34], could have impact on the susceptibility to skin cancer, the current evidence with the largest sample size to date provided a null outcome regarding the associations, for which the potential explanation is that a single polymorphism may have limited impact on the complicated pathogenesis of skin cancer than what we have anticipated. In support of this, the study by Schoof *et al*. [25] observed no significant association for skin cancer risk when considering a single polymorphism of IL-10, however, they found that the haplotype ITAGC from distal as well as proximal polymorphic site −7400In/Del, −6752A/T, −3538A/T, −1087G/A (−1082G>A), −597A/C (−592C>A) of the IL-10 was significantly associated with a reduced risk of developing melanoma. Hence, strategies based on more comprehensive haplotype or multiple polymorphisms rather than a single polymorphism may provide more precise information on genetic contribution of IL-10 to skin cancer aetiology.

With the employment of a meta-analysis approach, the present study is subjected to potential methodological deficiencies of the included studies and several specific limitations merit consideration. Firstly, the sample size for the studies polymorphisms seems to be insufficient to reach a reliable and confident conclusion, although appropriate analytical method were applied to minimize the potential bias. Secondly, the witnessed heterogeneity between studies in several comparisons may distort the results of the meta-analysis. Thirdly, our results are based on unadjusted estimates and a more precise analysis by adjusting some important compounding factors (such as age, cancer history, environmental factors, etc.) should be considered. Fourthly, all the included investigations in the current study were conducted in Caucasian population, which may limit the applicability and representativeness of results.

In conclusion, the present study suggested a potential association between IL-10 −819C>T polymorphism and decreased risk of skin cancer, but no association for −1082G>A and −592C>A polymorphisms. In light of the limited sample size and considerable limitations mentioned above, further large-scale multicenter epidemiological, multi-ethnicity-based studies considering comprehensive haplotype effect of IL-10 polymorphisms are urgently needed.

## METHODS

### Search strategy

Initially, an electronic search of PubMed, Embase, Web of Science, Chinese National Knowledge Infrastructure (CNKI), Wanfang and Chinese Biomedical Database was carried out to identify relevant studies up to June 2017 with the following search terms: “interleukin-10”, “IL-10”, and “skin cancer”, “skin carcinoma”, “melanoma”, and “polymorphisms”, “polymorphism”, “variant”, “genotype”. All the studies reported in English and Chinese were considered for screening. The bibliographies of identified studies and relevant review articles were further tracked manually and/or electronically for additional eligible studies. In case of duplication, only the study with largest sample size and sufficient information was selected.

### Selection criteria

Studies identified for inclusion subsequent to an eligibility evaluation were required to satisfy the following criteria: (1) studies concerning the associations between three common IL-10 polymorphisms(−1082G>A, −819C>T and −592C>A) and skin cancer risk; (2) studies with a case-control or cohort design; (3) all the cases with a pathological confirmation in the studies; (4) studies providing sufficient information on the genotype distribution in case and control groups or necessary information to calculate the odds ratios (ORs) and 95% confidence intervals (95% CIs).

### Data extraction

With a uniform reporting table, all eligible studies were carefully and independently reviewed by two investigators. Any disagreement was resolved by a consensus reached by all the reviewers. The following information was extracted from each study: first author, publication year, country, ethnicity, genotypes distribution in cases and controls, source of control, genotyping methods, cancer subtype and IL-10 polymorphisms.

### Statistical analysis

The strength of the associations between IL-10 polymorphisms and skin cancer risk were measured by the pooled ORs and 95% CIs under five genetic models (Allele, homozygote, heterozygote, dominant, recessive) using the RevMan 5.2 software (provided by The Cochrane Collaboration, Oxford, UK; http://www.cochrane.org/software/revman.htm). The significance of the association was determined by a *Z* test with a random or fixed effect model according to the between-study heterogeneity, and *p <* 0.05 was considered significant [35]. As for between-study heterogeneity, the Cochrane *Q*-test and I^2^ statistic test were performed to evaluate the heterogeneity assumption (*p >* 0.1 indicated the absence of heterogeneity) [36]. The fixed effect model was utilized to pool the individual OR and 95% CI with the absence of heterogeneity, otherwise a random effect model was applied. Potential publication bias was evaluated using the Begg's funnel plot and Egger's linear regression test by Stata12.0 software (Stata Corp., College Station, TX, USA) and *p <* 0.05 was considered as significant publication bias [37, 38].

## SUPPLEMENTARY MATERIALS TABLE


