# Supplementary material for: Synthetic analysis of associations between IL-10 polymorphisms and skin cancer risk
Source: Oncotarget. 2017 Dec 17;9(6):6728–36. doi: 10.18632/oncotarget.23385 (PMC5805509; doi:10.18632/oncotarget.23385)
Supplement: Supplementary file 1 [file oncotarget-09-6728-s001.pdf]

# Synthetic analysis of associations between IL-10 polymorphisms and skin cancer risk

## SUPPLEMENTARY MATERIALS

**Supplementary Table 1: Sensitivity analysis by sequential omission**

| Study omitted | CT+TT vs. CC model | | Effect model | CT vs. CC model | | Effect model |
|---|---|---|---|---|---|---|
| | *p* | OR [95% CI] | | *p* | OR [95% CI] | |
| Alamartine 2003 | 0.23 | 0.89 [0.73, 1.08] | F | 0.13 | 0.85 [0.70, 1.05] | F |
| Alonso 2005 | 0.05 | 0.82 [0.67, 1.00] | F | 0.03 | 0.79 [0.64, 0.97] | F |
| Gu 2008 | 0.02 | 0.78 [0.63, 0.97] | F | 0.02 | 0.77 [0.61, 0.96] | F |
| Howell 2001 | 0.15 | 0.86 [0.70, 1.06] | F | 0.13 | 0.84 [0.68, 1.05] | F |
| Martı´nez-Escribano 2002 | 0.05 | 0.83 [0.68, 1.00] | F | 0.03 | 0.80 [0.65, 0.98] | F |
| Nikolova 2007 | 0.03 | 0.80 [0.65, 0.98] | F | 0.07 | 0.82 [0.67, 1.02] | F |
| Sobjanek 2015 | 0.26 | 0.88 [0.72, 1.09] | F | 0.2 | 0.86 [0.69, 1.08] | F |

F, fixed effect model
